# Super-Genotype: Global Monoclonality Defies the Odds of Nature

**DOI:** 10.1371/journal.pone.0000590

**Published:** 2007-07-04

**Authors:** Johannes J. Le Roux, Ania M. Wieczorek, Mark G. Wright, Carol T. Tran

**Affiliations:** 1 Department of Tropical Plant and Soil Sciences, University of Hawaii at Manoa, Honolulu, Hawaii, United States of America; 2 Department of Plant and Environmental Sciences, University of Hawaii at Manoa, Honolulu, Hawaii, United States of America; Michigan State University, United States of America

## Abstract

The ability to respond to natural selection under novel conditions is critical for the establishment and persistence of introduced alien species and their ability to become invasive. Here we correlated neutral and quantitative genetic diversity of the weed *Pennisetum setaceum* Forsk. Chiov. (Poaceae) with differing global (North American and African) patterns of invasiveness and compared this diversity to native range populations. Numerous molecular markers indicate complete monoclonality within and among all of these areas (*F_ST_* = 0.0) and is supported by extreme low quantitative trait variance (*Q_ST_* = 0.00065–0.00952). The results support the general-purpose-genotype hypothesis that can tolerate all environmental variation. However, a single global genotype and widespread invasiveness under numerous environmental conditions suggests a super-genotype. The super-genotype described here likely evolved high levels of plasticity in response to fluctuating environmental conditions during the Early to Mid Holocene. During the Late Holocene, when environmental conditions were predominantly constant but extremely inclement, strong selection resulted in only a few surviving genotypes.

## Introduction

Biological invasions offer real-life situations of the roles of basic evolutionary processes, such as the essentiality of genetic diversity, the release from key limiting factors in native ranges and phenotypic plasticity in self-sustained population growth [1]–[2]. While general trends and characteristics of successful invasions remain for the most part elusive, many studies suggest that the ability to respond to natural selection might contribute more to invasion success than broad physiological tolerance or plasticity [reviewed in 3]. Introduced ranges typically represent sub-adapted environments akin to a “valley” on the adaptive topography; species without pre-adapted histories will only persist if plasticity allows genetic assimilation or if post drift allele frequencies allow for the adaptation towards an alternative fitness peak.

Founding plant populations often have depauperate genetic diversity and following range expansion, will generate low intra-populational genetic variation. This situation is exacerbated in self-pollinating and apomictic species with limited or no gene flow. As isolated selection and mutations are the only mechanisms for creating new alleles and variation, apomixes may represent an evolutionary dead-end [4], leading to genotypic diversity decay over time. Despite this, apomicts are frequently found as highly tolerant and successful invaders [5] supporting two of Baker's hypotheses [6]–[7]. First, apomictic species have the opportunity for a single propagule to colonize and spread into new environments, satisfying Baker's rule [7]. Secondly, many apomicts show developmental and phenotypic plasticity with broad environmental tolerances, conforming to a “general-purpose-genotype” as coined by Baker [6].

The allopolyploid apomictic grass weed, *Pennisetum setaceum* Forsk. Chiov. (fountain grass) was used to study whether global invasion gradients are shaped by genetic differentiation between areas that are differentially impacted by this plant. This species' native range spans parts of the Middle East and North Africa and as a popular ornamental plant it has escaped cultivation and invaded areas of Australia, Democratic Republic of Congo, Fiji, Hawaii, continental USA, Namibia, South Africa, Swaziland, Zambia and Zimbabwe [8]. We quantified neutral and quantitative genetic diversity of globally invasive and native fountain grass populations to determine the importance of such variation in invasion success.

## Results and Discussion

### Molecular genetic diversity

**Figure 1:**
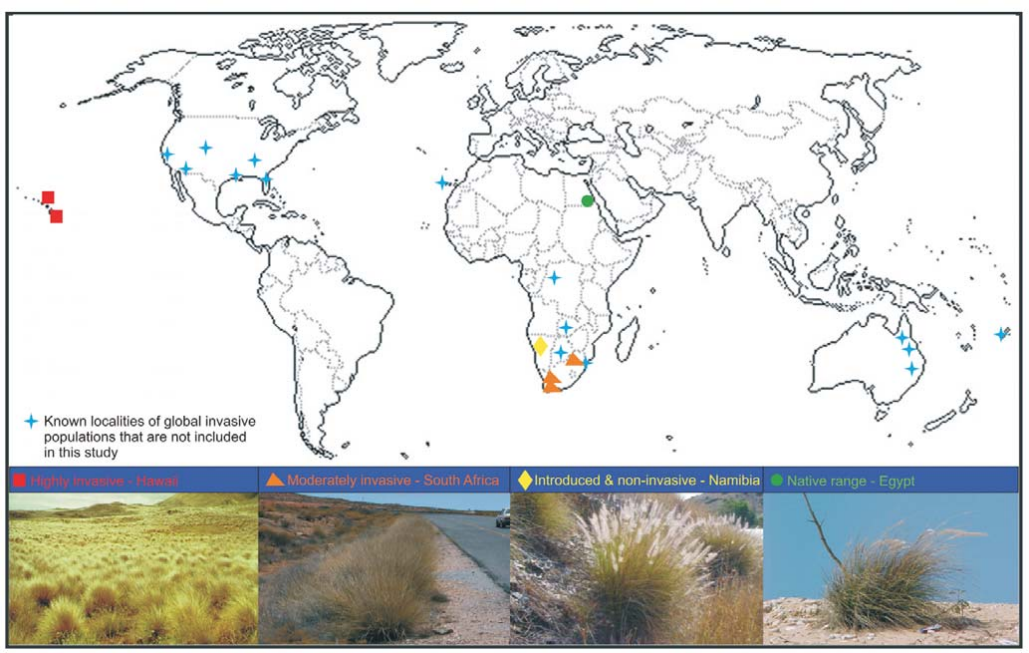
Global locations where differentially invasive and native range populations of *Pennisetum setaceum* were collected for this study. Additional world locations where fountain grass has been introduced and are considered invasive are also indicated.

**Figure 2:**
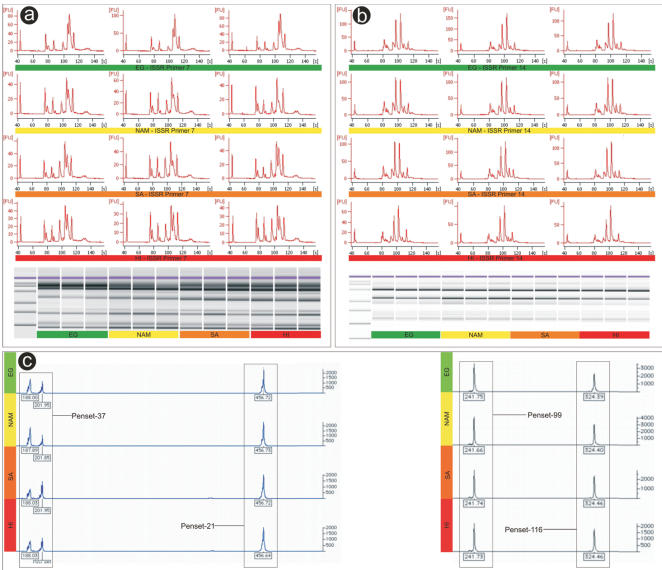
Results for selected molecular markers. ISSR banding pattern data for primer 7 (a) and primer 14 (b) indicating no variation between Egypt (EG, green), Namibia (NAM, yellow), South Africa (SA, orange) and Hawaii (HI, red). Electropherograms and their associated gel images are shown for 3 individuals from each of these locations. An illustration of 4 selected microsatellite loci (c) shows complete fixation for all alleles between all locations.

DNA sequencing of the internal transcribed spacer (ITS) regions 1 and 2 showed that all populations from all geographical regions share identical haplotypes for both genes. ITS2 was furthermore heterozygous in all individuals with the two alleles differing by 1 bp transitions at positions number 38 (T and C) and 101 (A and G), fixed for all populations. This initial lack of phylogeographical differentiation within and among extremely invasive *Pennisetum setaceum* populations characterized by nearly monotypic stands and aggressive competitiveness (Hawaiian archipelago), moderately invasive populations confined to disturbed habitats (South Africa), introduced but non-invasive populations limited to roadsides (Namibia) and native range populations (Egypt) (see Fig. 1) are indicative of an apomictic breeding system. We therefore turned our attention to molecular markers rendering higher intra-specific resolution. In general, genetic polymorphism is anomalously high for plant species with no known reproductive alternative(s) to apomixes and could be the result of mutations and/or multiple origins of clones [for e.g. see 9]–[11]. Despite this generalization, the subsequent usage of “high resolution” markers (microsatellites and inter simple sequence repeats [ISSR's]) failed to resolve any further differentiation. The 19 species-specific and diverse (length and motive class, Table 1) microsatellite markers had all alleles fixed across 320 individuals from all sampled locations (Table 2) (Fig. 2), indicating a single genotype shared globally and a panmixic *F_ST_* value of 0.0. Microsatellite sequences used as primer binding sites (ISSR's) supported all other molecular data in resolving a single genotypic fingerprint for all populations investigated (Fig. 2).

**Table 1:** Characteristics of the 19 microsatellite markers developed and used in this study.

Locus name	Forward/reverse primer sequences (5′→3′)	Repeat unit	Annealing temperature	Genbank accession number
Penset4	TATGGTTCGCCACTTGGTGC/ACCCTCTCACACCCTGGGAG	(GA)_18_	48.0°C	DQ899151
Penset6	CATATTTCAGACCGGGAACACC/AGGTCAGGGTCTCGGGTCG	(TC)_23_	60.0°C	DQ899152
Penset14	TGTCACCAATGGAGTTGCTC/GCGTATGTGGGTGTGTTGC	(AAC)_6_	58.5°C	DQ899153
Penset18	TCACTTTTGTGCCAGACTGC/TCAGCAGCTTGTGGCCCAC	(CT)_24_	50.0°C	DQ899154
Penset21	TTGGGATGGTGTGGACACC/ACCAAAGGATAAATCTCGCTGC	(TA)_7_(AC)_6_	48.5°C	DQ899155
Penset24	TCCTCACTCTTGCTCTCACG/CCCACATAGTTTGCGGTAGG	(CT)_15_	49.0°C	DQ899156
Penset28	GTGGTCTAACCGCCGATTAG/ACTAGCCAAACTTGGTTGATCG	(GA)_13_	50.0°C	DQ899157
Penset35	GCGAGCCTAACAGCGTTTC/CTCGTGTGGGCAGCAATGC	(GA)_33_	56.2°C	DQ899158
Penset37	TTGACGGGAAGAGCAAAGC/TGAATCGAGCCCAGGCTGC	(CT)_12_	52.1°C	DQ899159
Penset95	GGAGTGCTTGGAGACTTGC/CCAAATGGTACATACTAGCGGTTC	(GTT)_17_	53.0°C	DQ899160
Penset99	GCAATCAACGTGCCTGAACC/ATCCAGTGCCAGAGGCTCC	(GTT)_7_	48.5°C	DQ899163
Penset104	TGTTTCAGTCATGGGCTGAC/GCTTGCGATTGGGTCCTGAG	(CAA)_18_	60.5°C	DQ899165
Penset105	AGCAATTAGTGTGCCTGTAACC/TTTGCCACCAGCCGAGAGTC	(GTT)_7_	45.5°C	DQ899166
Penset110	CAATGTGTCTGAACCATGACCTC/AGCCTTTTGTCCCAAGCAAG	(GT)_8_(GGGG)(GT)_12_	60.0°C	DQ899168
Penset111	TGGGGTTGTCCTGGGGTGG/TGAGGAAGACAAAGCAATCACC	(GTT)_27_	50.5°C	DQ899169
Penset114	ACCCCAACTTGCTTGGGAC/TCTACGAGGACGCCTGTGG	(GTT)_6_	48.0°C	DQ899170
Penset117	CGCCATGCAACACAAGCAC/TCAAAGTGGTTGAGGGTTGC	(AAC)_21_	60.0°C	DQ899171
Penset119	TCACGTCGTAACAATGCACC/TGCTCAGGTGACTGCTCTG	(CAA)_8_	60.0°C	DQ899172
Penset120	ACAATCCCTGTGCCCAAAC/AGCTATCAACGTGCTTGAACC	(AAC)_6_	49.0°C	DQ899173

**Table 2:** Globally invasive and native populations of *Pennisetum setaceum* used in this study.

Origin (country)	Region*	Habitat/Vegetation	Latitude, Longitude
*Native range*			
Egypt	Medan Gohainah*	Desert sand dune/Sparse steppe	N 30.00825°, E 30.98019°
Egypt	Al-Geiza*	Desert sand dune/Sparse steppe	N 29.99233°, E 30.98936°
*Introduced and non-invasive*			
Namibia	Windhoek	Highland Savannah/Trees and grassland	S 22.58327°, E 016.97473°
Namibia	Windhoek	Highland Savannah/Trees and grassland	S 22.58243°, E 016.97683°
Namibia	Windhoek	Highland Savannah/Trees and grassland	S 22.58243°, E 016.97682°
*Moderately invasive*			
South Africa	Northern Cape*	Semi-desert/Succulent, shrub and grassland	S 30.47577°, E 017.95216°
South Africa	Northern Cape	Semi-desert/Succulent, shrub and grassland	S 30.47893°, E 017.94686°
South Africa	Western Cape*	Mountain Fynbos/Woody tree and shrub fynbos	S 31.94066°, E 018.69687°
USA	California	Mountain grassland	N 33.97183°, W 117.72305°
USA	California	Shrubland	N 34.10388°, W 118.6025°
*Highly invasive*			
USA	Kona, Hawaii*	Semi-arid/Dry forest, shrub and grassland	N 19.81157°, W 155.97464°
USA	Kona, Hawaii	Semi-arid/Dry forest, shrub and grassland	N 19.73449°, W 155.53534°
USA	Kona, Hawaii	Semi-arid/Dry forest, shrub and grassland	N 19.73663°, W 156.03279°
USA	Lanikai, Oahu*	Semi-arid/Shrub and grassland	N 21.46888°, W 157.74333°
USA	Lanikai, Oahu	Semi-arid/Shrub and grassland	N 21.47111°, W 157.735°

* Populations that were used for quantitative trait variance analysis.

Previous work [12] showed a similar lack of genetic differentiation for a similar invasive gradient of North American fountain grass populations from Hawaii (extremely invasive), Arizona (moderately invasive) and California (less invasive) using these same ISSR markers. Most plant introductions to Hawaii originate from the continental US and considering the relative geographical scale of Poulin et al.'s [12] study, the monoclonality observed could be the result of a single initial introduction event of fountain grass to the USA. However, based on our ISSR markers findings, we conclude that *P. setaceum* from South Africa, Namibia, Egypt, Hawaii, Arizona and California share a single genotype, supporting monoclonality on a much larger, inter-continental scale. Not surprisingly, additional genotyping of individuals from two Californian populations also proved that all fixed microsatellite alleles were shared with all other global populations investigated. More convincing of global monoclonality is that this single genotype also conforms to native Egyptian populations. Coupled with the broad native range of this species the Egyptian populations included in this study are unlikely to be the direct sources of the current globally introduced fountain grass populations and do not explain the sharing of a single genotype.

To overcome the evolutionary constraints of agamic reproduction most apomictic taxa are facultative apomicts, and sexual populations, even though rare, allows for the acquisition of genetic diversity [e.g. 13]–[14]. For example, Schmelzer and Renno [15] indicated substantial genetic variation in two agamic *Pennisetum* species (*P. subangustum* and *P. polystachion*) within their native African ranges. These species are closely related to fountain grass (tribe: *Paniceae*) illustrating that our findings are potentially specific to *P. setaceum* and do not reflect the consequence(s) of a broader taxonomic pattern. High levels of genetic diversity have also been demonstrated for other apomictic grass species in both native (e.g. *Heteropogon contortus*, Carino and Daehler [16]) and introduced (e.g. *Phragmites australis,* Saltonstall [17]) ranges. More recently, Lavergne and Molofsky [18] illustrated that, as a result of recombination and reshuffling within multiple and genetically diverse founding populations, invasive reed canarygrass (*Phalaris arundinacea*) had higher genetic diversity in its introduced ranges than in its native range.

### Quantitative genetic diversity

The widespread global distribution of fountain grass exposes it to unique and divergent selection pressures and that prompted us to measure ecologically meaningful fitness correlates (biomass allocation) to different environmental gradients (drought, nitrogen, total nutrients and soil pH) for Hawaiian, South African and where possible, Egyptian populations. Our choice of treatments was based on the marked differences or similarities for these abiotic components between our study regions. For example, in South Africa moderately invasive populations were found in both summer (monsoonal) and winter (polar front) rainfall areas that pose unique nitrogen fluxes, whilst highly aggressive Hawaiian populations inhabit volcanic ash soils that are typically poor in nitrogen. Furthermore, in South Africa, invading populations can be found in different biomes including temperate coastal Fynbos, sub-tropical Savannah, arid Succulent Karoo and Nama Karoo regions [19], representing diverse and distinctive environmental conditions. Measures of fitness correlates were useful in two ways: 1) given the lack of molecular genetic diversity we assessed the degree of phenotypic plasticity and whether adaptive variation exists for plasticity and 2) to partition quantitative genetic differentiation among these regions, thereby allowing comparison between neutral genetic variation (*F_ST_*) to non-neutral adaptive genetic variation (*Q_ST_*). Quantitative traits vary continuously owing to their polygenic nature and environmental influences, and coupled with selection strength, determine evolutionary potential. A strong correlation exists between quantitative trait variation (*Q_ST_*) and that observed for neutral markers (*F_ST_*) with *Q_ST_* typically exceeding *F_ST_* in natural populations [20]. This is consistent with the interpretation that polygenic traits are under directional selection varying in magnitude and direction as a function of differential selection pressures among different populations in different environments.

**Figure 3:**
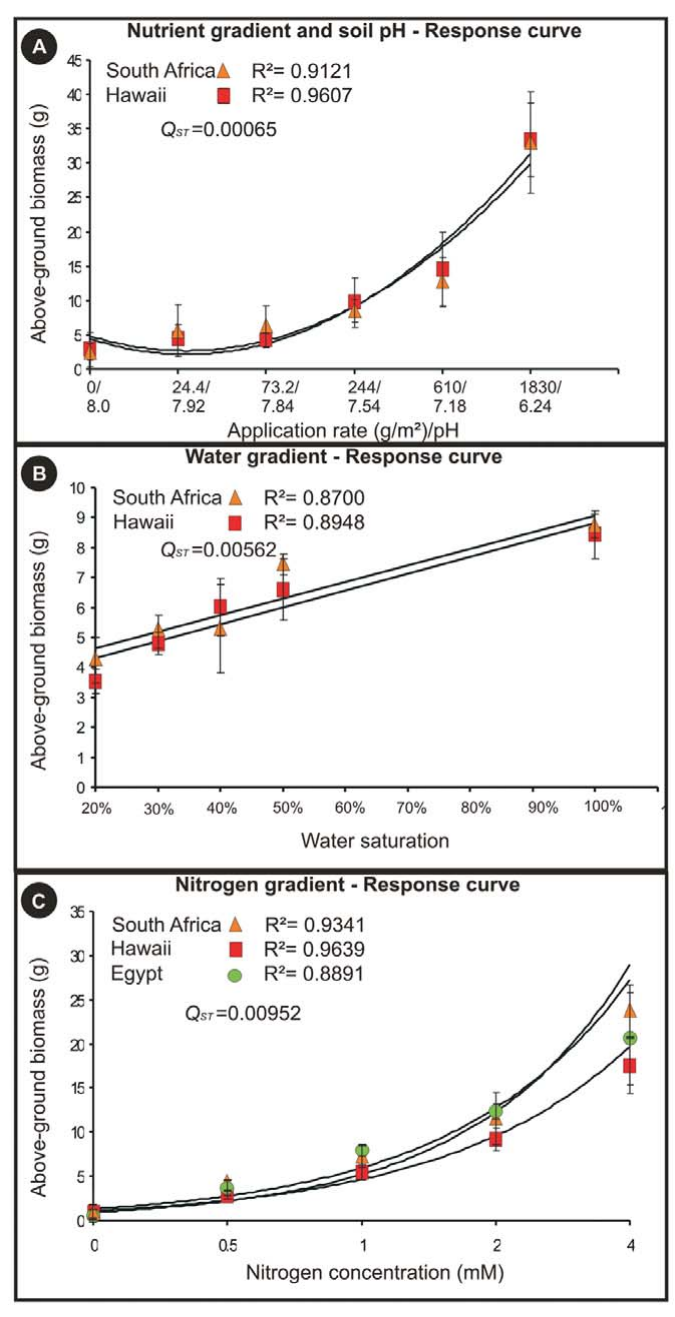
Genotypic reaction norms for (A) nutrient and soil pH gradient, (B) water gradient and (C) nitrogen gradient showing mean (±s.e.m.) biomass accumulated in response to each treatment level. Egyptian populations were only included for the nitrogen treatments. The corresponding *Q_ST_* values for fitness correlates are indicated on each graph.

Consistent with our molecular data, less than 1% of the variation was explained among differentially invaded (South Africa and Hawaii) and native range (Egypt) populations for fitness correlate measurements in response to the nitrogen gradient (*Q_ST_* = 0.00952). The same result was observed for differentially invaded areas (South Africa and Hawaii) in response to the nutrient and soil pH gradient (*Q_ST_* = 0.00065) and the water gradient (*Q_ST_* = 0.00562) (see Fig. 3 for genotypic reaction norms). Phenotypic plasticity was apparent for all populations investigated and a lack of additive genetic variation in plasticity was reflected in the similarity of the slopes and intercepts of the genotypic reaction norms (Indicator Variable Analysis; nutrient and soil pH gradient [F = 0.84; P = 0.362], water gradient [F = 0.16; P = 0.687] and nitrogen gradient [F = 0.42; P = 0.661], Fig. 3). Egyptian populations were only included for one of the treatments measuring ecological fitness correlates (nitrogen gradient) as all experimental procedures were terminated prior to plants flowering (seeding stage) and limited availability of seed stock. Despite the fact that native Egyptian populations were only included in one experimental treatment measuring quantitative genetic variation we feel confident that the results support overall monoclonality. Nitrogen sources (such as nitrate) have been shown to not only affect the expression of genes involved in nitrogen metabolism but also those involved in carbon metabolism [21]. Recently, a genome wide microarray analysis showed that nitrate alone affected the expression of 208 different genes in *Arabidopsis thaliana* [22]. Here, only 0.95% of the variation in such gene expression (possibly hundreds of genes involved in carbon and nitrogen metabolism) that effects biomass accumulation was accounted for among differentially invaded and native regions.

As with the adaptive evolution of any trait, polyphenism requires genetic variation in, and selection on that variation. Considering the geographical scale of our study, the lack of variation in ecological meaningful fitness correlates and adaptive plasticity is surprising. Most studies do reveal genetic variation in plasticity, even over very small spatial scales [23] and coupled with the known complexity of developmental plasticity, underscores the uniqueness of our findings. Our data were furthermore predisposed to maternal effects as treatments were started from seed directly collected in the field from all locations and were not subjected to a common environment prior to experimental manipulation making these results even more intriguing. *Pennisetum setaceum*'s native range populations and those introduced globally (in most instances more than a century ago) seemingly have undergone no local adaptation for developmental traits related to water, nitrogen or nutrient stresses, as adequate levels of plasticity guaranteed broad ecological range tolerance and fitness.

### Evolution of phenotypic plasticity in P. setaceum

Selective evolution of plasticity for any given population will in part depend on whether the plastic response has high energetic, functional and/or genetic costs [24]. Due to the lack of phenotypically more or less plastic individuals estimates of such costs were impossible to obtain during this study. Although plasticity costs are likely to constrain the evolution of plasticity for a given trait, the ubiquity of plasticity in many species suggests that the benefits outweigh the costs under a wide variety of conditions. Costs related to plasticity do, however, prevent the continued evolution of plasticity to the point where a species could be successful in most environments [25]. Opposing this general perception, the single genotype described here supports the hypothesis that plasticity is the sole mechanism driving this genotype's success in heterogeneous and novel environments. Similarly, Williams and co-workers [26]–[27] found that high levels of plasticity exist in ecophysiological traits in reciprocally transplanted fountain grass populations conforming to an altitudinal gradient spanning sea level to sub-alpine habitats in Hawaii. Without sufficient plasticity, rapid changes in conditions and environments such as these to which *P. setaceum* may never have been exposed, will pose a particular risk of local extinction. Costs associated with maintaining such high levels of polyphenism are expected to trigger the evolution of reaction norms that facilitates adaptation to more frequently encountered environments [28]. Therefore, we postulate that the single genotype described here was historically extremely cost-effective even when multiple plastic hybrid genotypes likely existed. For the evolution of plastic genotypes such as the one reported here, single genotypes or genotypes over a few generations must be subjected to heterogeneous environmental condition exposures. However, this situation still does not explain why apparently only one genotype exists. To explain this, we furthermore speculate that at some point native genotypes of *P. setaceum* were exposed to constant environmental conditions that were extremely hostile and caused hard purifying selection, depriving these populations of genetic variation to very low or possibly non-existing levels, allowing only for the most plastic genotypes to persist.

Numerous paleontological records attest that the Late Pleistocene epoch was characterized by arid conditions accompanied by aeolian activity in Egypt and Sudan. Aridity abated during the Early to Middle Holocene as a transition towards episodic humid and wetter conditions punctuated by intervening arid phases occurred with primarily steppe vegetation covering aeolian sands [29]–[31]. These fluctuations, corresponding to Indian Ocean monsoon (IOM) intensity oscillations resulting from glacial boundary forcing (e.g. sea surface temperatures), occurred on timescales as short as decadal to multidecadal [32]. *Pennisetum setaceum*'s agamospermous reproductive system likely assured single genotypes' preservation and thus exposure to such fluctuations over the course of multiple generations. These variable conditions in the ancestral environment would have favored the evolution of high levels of phenotypic plasticity. Review of botanical evidence from the Eastern Sahara furthermore suggests that the grasslands in Egypt and Sudan were diminishing around 7000 BP when these fluctuating climate conditions waned and constant aridity began to set in, dramatically reducing the desert flora to a similar composition to the present day flora [33]. Aridification led to the establishment of arid-to-hyperarid conditions across the region by ∼ 4500 BP with the extant flora disappearing from most habitats, and becoming restricted to only the hardiest desert-adapted plants [34]. Such inclement environmental conditions would have exerted extremely strong selection on ancestral populations of *P. setaceum* and might have allowed only a few of the most plastic genotypes to explore the new adaptive landscape and to survive.

In addition, an agamospermous reproductive system would have stabilized hybridity and thus conserve this seemingly well-adapted genotype(s) of *P. setaceum*. Two possible attributes could have further contributed to the success of this single allotriploid genotype since its prehistoric origin. First, stabilized hybridity also leads to fixed heterotic genotypes and boosts fitness as afforded by fixed heterozygosity. Secondly, this fixed heterozygosity leads to a dumping of genetic load by masking detrimental mutations accumulated by the pre-hybridization parental lineages.

Plasticity is essential for populations to persist in novel environments and following establishment, heritable differences can be accumulated by natural selection to the extent where the adaptive phenotype achieved via plasticity becomes genetically fixed [35]. However, traits that are highly plastic are unlikely to be subject to divergent selection and may not become genetically divergent from the source [36]. Extremely high levels of plasticity enabled *P. setaceum* to cross adaptive valleys on the adaptive topography (analogous to conditions historically not encountered) to reach alternative optimal fitness peaks without the need for local adaptation and thus the chance to differentiate from source populations. *Pennisetum setaceum* is thus a classic, but extreme example of a general-purpose genotype [6]. Generality is fundamental for apomictic survival as clones surviving for many generations could only have done so by virtue of being able to tolerate all environmental variation exposed to since their origin, while more specialized genotypes would have rapidly gone extinct. Here, we provide evidence for the existence of a single, successful apomictic genotype on a global scale showing wide environmental tolerance and propose the term super-genotype to define this unique phenomenon. A super-genotype for *P. setaceum* is justified by the apparent lack of neutral and adaptive genetic differentiation within the region of origin and among globally introduced populations and the grass' capability to survive under a wide array of environmental conditions. High levels of phenotypic plasticity ensure self-sustainability in disturbed habitats [37] but do not seem to allow for fountain grass to overcome the biological resistance-buffer posed by undisturbed habitats and intact ecosystems. The gradient of invasiveness observed here is indeed correlated to some degree to a similar disturbance-level gradient between differentially invaded areas. Namibian fountain grass populations are only found in human-derived disturbance areas such as roadsides with no spread or establishment in undisturbed native vegetation [8]. In South Africa, however, where limited spread and persistence in native vegetation does occur [19], ecosystems are characterized by intermediate disturbance levels. In these Mediterranean-climate shrubland (Fynbos) areas fire disturbance often yields spatial heterogeneity and intermediate environmental disturbances [38]. The islands of the Hawaiian archipelago's, and in particular the island of Hawaii's, geological recentness (0–0.5 Ma) encompasses habitats that are overall in a constant state of extreme disturbance [39]. Even though this genotype is well-adapted to disturbed habitats, evolutionary potential is essentially impossible and any fitness peak or slope thereof that is out of reach to its plastic response will result in rapid extinction.

### Conclusion

Understanding the underlying processes and variables that affect a population's ability to adapt and survive in changing and/or novel environments are critical issues in evolutionary biology, conservation biology, and ecology [2], [40]. In contrast to typical Darwinian evolution, the single super-genotype identified here persists and survives exposure under most environmental conditions. Further examination of other species may reveal further super-genotypes, and it may be found that this is a more common, significant but hitherto overlooked mechanism driving survival and local fitness of plant populations.

## Materials and Methods

### Population sampling

Leaf material and where possible seeds were randomly collected from 20–30 individuals per population of *P. setaceum* in South Africa, Namibia, Hawaii, California and Egypt (Table 2).

### DNA extraction and sequencing of Internal Transcribed Spacer Regions (ITS1 and ITS2)

Genomic DNA was extracted with the DNeasy Plant mini kit (Qiagen, Germantown, USA). The entire ITS region was PCR amplified using conditions and primers described by Martel et al. [41]. Each reaction contained 5 ng of total genomic DNA, 0.4 (v/v) HotMasterMix (HotMaster Taq DNA polymerase, 0.3 U; 2.5× HotMaster Taq Buffer pH 8.5, 45 mM KCl and 2.5 mM MgCl_2_; 200 µM of each dNTP [Brinkman Instruments Inc., Westbury, USA]) and 25 pmol of both primers. Purified PCR products were sequenced in both directions and were run on an ABI377 automated sequencer (Applied Biosystems, Foster City, USA.) using standard dye-terminator chemistry. DNA sequences were manually aligned and submitted to GenBank (AY944426-AY944463).

### Microsatellite libraries and marker development

We developed microsatellite enriched-libraries as described by Hamilton et al. [42] with slight modifications. Fifty micrograms of extracted genomic DNA were digested with *Rsa*I, *Hae*III, *Alu*I and *Hinc*II and the resulting fragments simultaneously ligated onto SNX oligonucleotide linkers. Following enrichment with biotin-labeled microsatellite oligoprobes (AAC_8 _and GC_12_) and streptavidin-coated magnetic beads, PCR amplification and purification, putative microsatellite-containing fragments were digested with *Nhe*I, and ligated into the *Xba*I site of pUC19 prior to transformation.

Transformed bacterial colonies were transferred onto nylon membranes and screened using digoxigenin labeled microsatellite probes to identify microsatellite-containing clones. Following DNA extraction from clones, vector insert fragments were PCR amplified, purified and sequenced. DNA sequences were visually inspected to identify perfect and compound microsatellite sequences and PCR primers specific to each locus designed using the program fastpcr [43].

### Screening for polymorphism and genotyping

Initial screening for polymorphisms was done by analyzing alleles for 6 different individuals from each global region (total of 24 individuals). PCR reactions were carried out in 15 µL total reaction volumes. Each reaction contained 0.4 (v/v) HotMasterMix (HotMaster Taq DNA polymerase, 0.3 U; 2.5× HotMaster Taq Buffer pH 8.5, 45 mM KCl and 2.5 mM MgCl_2_; 200 µM of each dNTP [Brinkman Instruments Inc., Westbury, USA]), 7.5 pmol of each primer and approximately 5 ng total genomic DNA. A thermocycle of an initial denaturation of 94°C for 2 min followed by 35 cycles of 1 min at 94°C, 1 min at the appropriate annealing temperature (Table 1) and 1 min at 72°C, followed by a final 12 min extension at 72°C. Purified PCR products were run on an Agilent 2100 Bioanalyzer analysis LabChip (Quantum Analytics, Inc., Foster City, CA) to detect fragment size differences at each locus.

Loci were also genotyped by fluorescently labeling PCR products using PCR cycle conditions described above and fluorophore-labeled primers. Separation of specific alleles was carried out on 5% polyacrylamide gels using an ABI377 sequencer. Analysis of the gels and the fragment lengths were carried out using the software GeneMarker (Softgenetics LLC^TM^, PA). Fragments of lengths 50–700 bp were manually scored.

### Inter-simple sequence repeat marker (ISSR) diversity

ISSR diversity was assessed using primers described by Poulin et al. [12]. Each PCR reaction contained approximately 2 ng of total genomic DNA, 0.4 (v/v) HotMasterMix (HotMaster Taq DNA polymerase, 0.3 U; 2.5× HotMaster Taq Buffer pH 8.5, 45 mM KCl and 2.5 mM MgCl_2_; 200 µM of each dNTP [Brinkman Instruments Inc., Westbury, USA]) and 50 pmol of ISSR primer. A thermocycle of an initial denaturation of 94°C for 2 min followed by 35 cycles of 1 min at 94°C, 1 min at the appropriate annealing temperature (Table 3) and 1 min at 72°C, followed by a final extension of 12 min at 72°C. PCR products were run on an Agilent 2100 Bioanalyzer analysis LabChip (Quantum Analytics, Inc., Foster City, CA) to visualize and compare banding patterns for each ISSR marker.

**Table 3:** ISSR primers used in this study.

Marker name	Primer sequence	Annealing temperature
Primer 7	CACACACACACAGA	43.0°C
Primer 8	CTCTCTCTCTCTCTCTRG	43.0°C
Primer 10	GAGAGAGAGAGAGAGA	44.5°C
Primer 14	GTGTGTGTGTGTGTYG	50.0°C
Primer 16	GACGACGACGACRC	50.0°C
Primer 17	GTCGTCGTCGTCRC	48.0°C
Primer 18	GTGGTGGTGGTGRC	50.0°C

### Developmental traits

Twenty families from South Africa, Hawaii and Egypt were included for analysis of developmental variation under various conditions. Each family consisted of seedlings produced from seeds harvested from one of 20 randomly chosen individuals from each geographic location. Prior to all treatments, seeds were allowed to germinate and grow for two weeks in a 1∶1 vermiculite and potting soil mixture before being transplanted into 800 mL plastic pots. A total of 10 replicates from each geographical location, i.e. 10 randomly chosen families, were used in each treatment. Pots were randomly repositioned weekly in the greenhouse for the duration of all experiments. Responses to all different treatments were measured as the amount of above-ground biomass produced after a certain growth period. Egyptian populations were only included for the nitrogen gradient due to limited seed stock available and as all experimental procedures were terminated prior to plant flowering (seeding stage).

### Water Stress

Seedlings were maintained at five different levels of water availability representing well-watered (obtained by watering the potting soil and vermiculite mixture (1∶1) and allowing percolation to finish) to severe water stress (50, 40, 30, 20% of the maximum water retention capacity i.e., well-watered) conditions. Plants were watered with the appropriate amount of water each day. Above-ground biomass was harvested and weighed after 43 days.

### Nitrogen Availability

After transplanting seedlings in vermiculite a nitrogen gradient was simulated by applying complete micronutrient solutions with nitrate concentrations fixed at 0, 0.5, 1, 2 and 4 mM NO_3_^−^, all other elements applying to nitrogen-free nutrient solution concentrations [44]. Fifty milliliters of the appropriate nutrient solution was applied twice a week throughout the duration of the experiment to each pot. Plants were watered daily to keep vermiculate at saturated moisture content. Above-ground biomass was harvested and weighed after 60 days.

### Nutrient Availability

A nutrient gradient was created by applying slow-releasing fertilizer (Osmocote® [total nitrogen, 9.0%; available phosphate, 6.0%; soluble potassium, 6.0%; total sulphur, 18.7%; Iron, 2.0%]) at different rates (24.4 g/m^2^, 73.2 g/m^2^, 244 g/m^2^, 610 g/m^2^ and 1.83 kg/m^2^) to seedlings planted in a 1∶1 vemiculite:potting soil mixture. Plants were watered daily to simulate well-watered conditions. Above-ground biomass was harvested and weighed after 45 days. The slow-releasing fertilizer promoted acidic soil conditions, simultaneously creating a pH gradient. Soil pH was measured at the time of harvest after continuous stirring of 50 g of soil in 300 mL distilled water for 20 min.

### Analytical methods

A nested ANOVA [45] was used to partition and estimate variance components of developmental responses, and to estimate *Q_ST_* values as *Q_ST_* = V_o_/(V_o_+V_t_), where V_o_ is variance among families by geographical origin (country), and V_t_ is variance among families by treatment (nested within origin) [46]. *Q_ST_* values were calculated for the quantitative traits measured as accumulated biomass in response to water, nutrients and nitrogen availability.

Responses to treatments (water, nutrient and nitrogen availability) of plants from various origins were compared using linear regression [47]. To compare the effect of the qualitative variable “origin”, indicator variables were generated and full model multiple regressions (including indicator variables as independent variables) were compared to reduced models (with origins pooled) to determine if origin had any significant effect on the slope and intercept of the response curves. This analysis was conducted for each of the treatments separately.
